# LC-HR2FNet: High-Resolution Early-Level Fusion-Based LiDAR-Camera Network for Accurate Road Segmentation Autonomous Driving

**DOI:** 10.3390/s26113281

**Published:** 2026-05-22

**Authors:** Lele Wang, Ming Li, Peng Zhang

**Affiliations:** 1 School of Artificial Intelligence, Suzhou Chien-Shiung Institute of Technology, Taicang 215400, China; 9076@csit.edu.cn; 2 School of Computer Engineering, Jiangsu Ocean University, Lianyungang 222005, China; minglee2015@126.com

**Keywords:** sensor fusion, deep learning, point cloud, high resolution net, autonomous driving

## Abstract

Accurate road segmentation is a core perceptual technology for autonomous driving, but faces two challenges: (1) ambiguous road boundaries caused by insufficient modeling of contextual information relationships in CNN-based networks and (2) inadequate LiDAR-camera fusion due to modality gaps between heterogeneous sensors. To mitigate these limitations, this paper proposes a novel approach, named LiDAR-Camera High-Resolution Feature Fusion Network (LC-HR2FNet), a multi-cross-stage fusion model designed for road segmentation. Firstly, a new type of pseudo-LiDAR-Image representation is generated via an early-level fusion strategy and data complementation. Sparse point clouds are transformed into dense LiDAR-Image data and then concatenated with RGB channel maps to form complementary multi-modal data inputs. Subsequently, a modified HRNet backbone integrated with cross-stage feature fusion is constructed to strengthen information interaction across different branches and enhance the modeling of contextual relationships. Additionally, a dilated feature collection model is designed to collect multi-scale confidence scores for pixel-wise class determination. Experiments on the KITTI road benchmark demonstrate that the proposed method achieves a MaxF of 97.39% on UMM_ROAD and an average of 96.28% across all urban scenarios, demonstrating superior performance and robustness.

## 1. Introduction

Autonomous vehicles rely on the analysis of on-board sensor data to achieve robust environmental perception, which serves as a cornerstone for real-time navigation, decision-making, and motion planning, and is critical to ensuring the operational safety and reliability of intelligent vehicle systems [1]. Road segmentation, as a pivotal perception task, aims to semantically delineate each 3D point and pixel within the field of vehicles’ perception as either “road” or “non-road” areas. It primarily leverages two types of on-board sensors: RGB camera and Light Detection and Ranging (LiDAR) sensors [2], which provide information around the vehicle [3].

Research in road segmentation is broadly categorized by sensor technology into Camera-based [3,4,5,6,7,8,9,10,11,12], LiDAR-based [13,14,15,16,17,18,19], and multi-sensor fusion-based [20,21,22,23,24,25,26,27,28,29,30,31,32,33,34,35,36,37,38,39,40] methods. Camera-based methods can capture dense, texture-rich RGB images [4] with abundant semantic cues, enabling effective visual scene understanding [5]. However, such methods are inherently sensitive to illumination variations and dynamic environmental changes [6], and they fail to directly extract explicit 3D geometric depth information, which is a crucial need for autonomous driving applications [7,8,9,10,11,12].

Conversely, LiDAR sensors deliver precise 3D structural distance measurements and demonstrate robustness to lighting and weather conditions [13,14,15,16,17,18,19], yet the resulting point clouds are inherently sparse (and become sparser with increasing detection distance) and lack color and texture. Since each sensor modality captures complementary characteristics of surface items, fusing multiple data sources can yield a more comprehensive and reliable scene understanding than either modality in isolation for road segmentation.

Despite this potential, effective LiDAR-camera fusion remains a practical challenge, primarily due to the profound modality gap (3D sparse point clouds vs. 2D dense images) and the limitations of existing fusion strategies. Previous convolutional works [18,20,21,22,23] simply project the 3D data onto the image plane to establish cross-modal correspondences between 3D points and 2D pixels. However, this projection typically causes the loss of height and depth, resulting in an incomplete geometric representation of the scene. Fusion strategies are further divided into three levels: early-level, middle-level, and late-level fusion. Early-level fusion [20,21,22,23] concatenates raw data before processing, but suffers from interference between unprocessed heterogeneous data streams. Middle-level fusion [24,25,26,27,28,29,30,31] merges intermediate features, yet the selection of optimal fusion layers remains an unresolved question, which may cause information loss or redundancy. Late-level fusion [32,33,34,35,36,37,38,39,40] combines final predictions of separate modality-specific branches at the decision layer, but it neglects critical intermediate cross-modal feature interaction. Although middle and late fusion have been extensively studied in existing research, early-level fusion methods for LiDAR-camera road segmentation are still underdeveloped, leaving a critical research gap in the field. This paper aims to fill this research gap and further advance the understanding of road perception.

Deep learning techniques have driven significant progress in road segmentation, but mainstream CNN architectures suffer from information distortion with the increase in network depth, and they often ignore fine-grained boundary and edge details of road regions. Furthermore, previous methods fail to address the cross-stage feature gap between high- and low-resolution feature maps, which hinders the effective integration of primary features and supplementary contextual information. To tackle the above challenges, this paper proposes the LiDAR-Camera High-Resolution Feature Fusion Network (LC-HR2FNet), a multi-modal fusion architecture for road semantic segmentation. The framework contains two core components: Pseudo LiDAR-Image Data Generation and a modified HRNet with cross-stage feature fusion and dilated feature collection (HR2FNet). First, RGB images and sparse 3D LiDAR points are fused to generate a unified multi-modal representation as the HR2FNet input. Second, a cross-stage feature fusion module is integrated into the HRNet backbone to preserve high-resolution feature details, and a dilated feature collection module is designed to aggregate multi-scale confidence scores for pixel-wise road/non-road classification.

The main contributions of this work are listed as follows:

(1) A novel multi-cross-stage fusion framework (LC-HR2FNet) is proposed for LiDAR-camera road segmentation, which is the first architecture to integrate cross-parallel feature fusion and dilated multi-scale feature collection. The framework fills the research gap in early-level high-resolution fusion for heterogeneous sensor data.
(2) An early-level fusion strategy is designed to construct a high-resolution unified pseudo-LiDAR-Image representation (multi-channel RGBXYZ format), which realizes complementation of dense geometric cues from upsampled 3D data and rich visual textures from RGB images at the raw level. This strategy effectively bridges the modality gap between LiDAR and camera and minimizes cross-modal information loss.
(3) A modified HR2FNet with cross-stage feature fusion is developed to preserve high-resolution feature details and enhance the interaction of multi-scale intermediate features across different network branches. Additionally, a dilated feature collection module is introduced to aggregate sufficient confidence scores for pixel-wise class determination.

## 2. Related Work

Road segmentation in autonomous driving has been an active research topic for decades and is a fundamental task for robot applications. This section briefly reviews the related works, which are categorized into single-modality-based and multi-modal data fusion-based methods.

### 2.1. Single-Modality-Based Road Detection

Early road detection research focused on leveraging single-sensor data, mainly including camera-based and LiDAR-based methods. Traditional camera-based methods relied on hand-crafted features and visual boundary prior knowledge [7,8,9] to segment the road region. With the rapid development in deep learning, CNN-based methods [10,11,12] have become the mainstream for camera-based road segmentation, as they can automatically learn discriminative visual texture features from RGB images. SFNet [12] has been proposed to enhance semantic segmentation in low-light road scenes.MultiNet [13] proposed a unified framework to handle multiple perceptual tasks. However, camera-based methods are limited by the lack of 3D geometric information and high sensitivity to environmental changes such as illumination and shadow.

To overcome the limitations of 2D imagery, some researchers have turned to LiDAR data. For instance, Some works [14,15,16] explored the speed–accuracy trade-off for LiDAR-based boundary detectionand developed specialized solutions for challenging scenarios such as mining environments. RES3Dvelo [18] constructed a graph via Delaunay Triangulation using projective point clouds to capture road geometric structures. To overcome the inherent sensitivity of cameras to adverse conditions, Recently, advanced hybrid CNN-Transformer models [19] have been applied to further boost segmentation performance. Despite these advances, single-modality methods are inherently constrained by the physical limitations of individual sensors, leading to unsatisfactory performance in complex and dynamic driving environments.

### 2.2. Multi-Modal Data Fusion-Based Detection

Multi-modal data fusion strategies are becoming mainstream, which are broadly categorized into early-level [20,21,22,23], middle-level [24,25,26,27,28,29,30,31], and late-level fusion [32,33,34,35,36,37,38,39,40], each presenting distinct trade-offs in terms of alignment requirements and cross-modal interaction.

**Early-level fusion** aggregates raw sensor measurements into a joint representation before deep processing, which maximizes the preservation of cross-modal information. Wulff [20] projected LiDAR points onto the image plane to build a multi-dimensional occupancy grid encoding intensity, height, and reflectivity. Byeongjun et al. [21] converted both 3D data and image data into a bird’s-eye representation to facilitate efficient fusion or employed spherical-coordinate transformations [22] to reduce the dimensionality of LiDAR data for faster inference. More sophisticated strategies like LIF-Seg [23] have introduced coarse-to-fine early-fusion with an offset-rectification module to mitigate spatiotemporal misalignment. While effective, early fusion demands high-precision spatiotemporal calibration and can be susceptible to interference between unprocessed data streams.

**Middle-level fusion** extracts modality-specific features via separate encoders and then merges them within a deep neural network. Chen [24] calculated an altitude difference from LiDAR data and designed a cascaded fusion framework. Fan et al. [25] merged RGB images with depth information derived from stereo images, incorporating surface normal data [26] into the fusion process for free-space detection. Caltagirone et al. [27] incorporated RGB image data and an interpolated 2D LiDAR image [28] into a modified CNN. A major limitation is the lack of a clear criterion for selecting optimal fusion layers, and most methods rely on simple addition or concatenation operations for feature merging, leading to suboptimal cross-modal interaction. Recently, researchers have also explored replacing LiDAR data with depth maps to simplify the fusion process [29,30,31].

**Late-level fusion** combines the final predictions from separate modality-specific branches at the decision level, sometimes incorporating probabilistic modeling or mutual relationships. Conditional Random Field (CRF)-based methods [32,33,34,35] are prominent in this category. These methods either integrate CRF at the unary stage [32] or use it as post-processing for superpixel labeling [33]. Advanced hybrid CRF [34,35] models have been developed to incorporate interactions between 3D data, 2D images, and cross-modal links. Moreover, Gu et al. [36] developed a depth-induced fusion framework; another approach [37] generates depth images via joint bilateral filters, and refines outputs with CRF; Gu et al. [38] developed a height-difference-based method to blend dual-modal outputs (with an energy function including 2D/3D unary and 2D–3D pairwise potentials) and later built on [36] to propose an improved Delaunay triangular upsampling strategy [39] by accounting for projection point distribution. However, these approaches often face challenges related to the imbalance caused by sparse LiDAR data and may neglect crucial intermediate cross-modal semantic interactions. Feng proposed to combine evidence from two modalities using an uncertainty-aware fusion module [40].

Early fusion inherently demands high-precision spatiotemporal calibration. However, a critical gap persists in designing simple yet effective early-level fusion modules that can reduce data complexity while enabling meaningful cross-modal feature interaction. This work aims to address this gap by proposing a unified data representation and a high-resolution fusion network.

## 3. LC-HR2FNet Method

**Figure 1:**
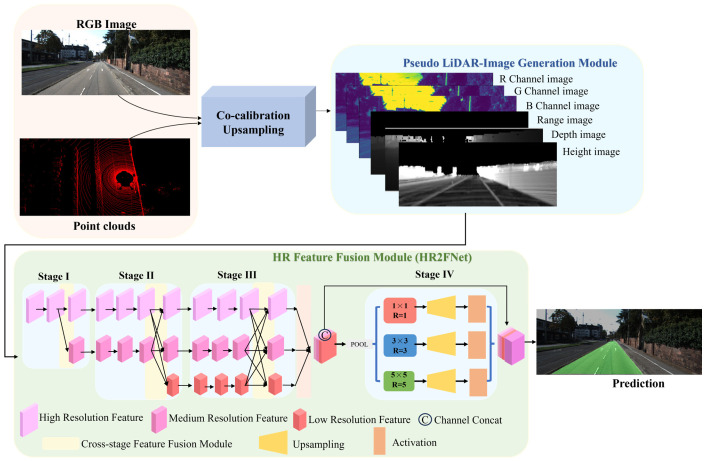
The flowchart architecture of the LC-HR2FNet framework. Specifically, the framework takes RGB images and LiDAR point clouds as inputs. First, in the pseudo-LiDAR-Image generation module, sparse 3D points are projected onto the image plane and further **densified** to construct dense LiDAR-image data. These geometric features are then **concatenated** with RGB channel maps to form a **6-channel unified multi-modal input**. Next, the HR2FNet backbone extracts multi-scale features, in which the **cross-stage feature fusion module** enhances information interaction across branches of different resolutions. Finally, the **dilated feature collection module** aggregates multi-scale confidence scores and outputs the final pixel-wise result.

The overall framework of the LiDAR-Camera High-Resolution Feature Fusion Network (LC-HR2FNet) is illustrated in Figure 1, which takes synchronized RGB images (from RGB camera) and sparse 3D point clouds (from Velodyne 64E LiDAR) from the KITTI Road benchmark [41] as input. The framework has two core components: the Pseudo LiDAR-Image Data Generation and the HR2FNet. The former step involves an early-level fusion strategy and upsampling data complementation operations to acquire dense LiDAR-Image data, and through concatenating RGB channel maps to generate a six-channel pseudo-LiDAR-Image data. In the latter, a modified HRNet backbone integrated with a cross-stage feature fusion module and a dilated feature collection module, which extracts high-resolution multi-scale features from the pseudo-LiDAR-Image input and aggregates multi-scale confidence scores for precise pixel-wise road/non-road classification.

### 3.1. Pseudo LiDAR-Image Data Generation

Given RGB images $R\epsilon R^{H\times W\times 3}$, and a sparse LiDAR point cloud $P\epsilon R^{N\times 4}$, the pseudo-LiDAR-Image data generation aims to transform sparse 3D data into a dense, geometry-rich form that complements the RGB image for road perception. It consists of an early-level fusion strategy and a dense data complementation module.

In the former, 3D point data are projected onto the 2D image plane via extrinsic and intrinsic parameters to establish cross-modal correspondence between RGB-based visual information and LiDAR-based geometric data. In the latter, to mitigate the limited data sparsity and loss of height/depth information during projection, the dense data complementation module is introduced, which is the core improvement of the data generation step.

Finally, the R, G, B channel maps are concatenated with the dense LiDAR-Image to form a six-channel pseudo-LiDAR-Image data representation.

**Figure 2:**
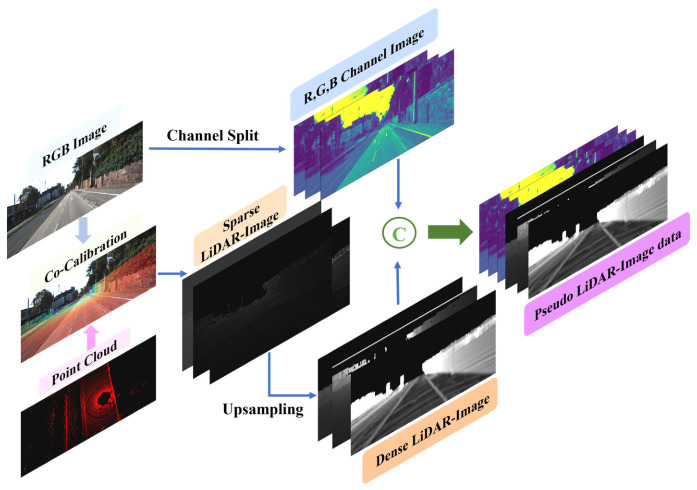
Schematic of pseudo-LiDAR-Image data generation.

The resulting multi-channel representation integrates visual texture with dense geometric cues from the dense LiDAR data, effectively bridging the modality gap between heterogeneous sensors. The detailed flowchart process is illustrated in Figure 2.

#### 3.1.1. Early-Level Fusion Strategy

3D data and RGB images span distinct dimensional spaces; a critical step is to unify their representations within a shared feature space. Early-level fusion strategy converts the points in a 3D coordinate system into a 2D grid-like structure that aligns with the spatial layout of the RGB image.

(1) $$P_{cam}=R_{rect}^{0}\cdot T_{velo}^{cam}{\cdot P}_{lidar}$$

(2) $$p_{cam}=T_{proj}\cdot P_{cam}$$

(3) $$T_{velo}^{cam}=\left[\begin{array}{cc} R_{velo}^{cam} & t_{velo}^{cam}\\ 0 & 1 \end{array}\right]$$

where $R_{rect}^{0}$ is a rectification rotation matrix, $t_{velo}^{cam}$ is a transformation matrix, and $T_{proj}$ is a projection matrix. Points with negative Z-value and points projected outside the field of view are discarded in the processing step.

#### 3.1.2. Dense Data Complementation Module

**For the LiDAR-image height map**, the height difference transformation [24] is introduced to calculate the value $H_{x,y}$ at (x,y). A dense height map can be obtained by calculating the absolute value of the spatial offset between two positions. The formula is defined as

(4) $$H_{x,y}=\frac{1}{M}\sum_{N_{x},N_{y}}\frac{\left|Z_{x,y}-Z_{N_{x},N_{y}}\right|}{\sqrt{{\left(N_{x}-x\right)}^{2}+{\left(N_{y}-y\right)}^{2}}}$$

where $z_{x,y}$ denotes the height value of the corresponding 3D data projected onto (x,y), $N_{x}$ and $N_{y}$ present positions of points (x,y), and M is the total number of neighborhood pixels.

**For LiDAR-Image depth and range maps**, the bilateral filtering is adopted to calculate each pixel, and the output image $D_{p}$ is given as follows:

(5) $$D_{p}=\frac{1}{W_{p}}\sum_{q\in N}G_{\sigma_{S}}\left(∥p-q∥\right)G_{\sigma_{r}}\left(\left|I_{q}\right|\right)I_{q}$$

where I is a sparse depth map, $I_{q}$ is depth information from 3D data at position q. N represents the spatial domain. $W_{p}$ is a normalization factor to ensure that the sum of weights equals 1, so that the converted grayscale value ranges from 0 to 255. $G_{\sigma_{s}}$ is a distance penalty term, inversely proportional to Euclidean distance ($\left(\left\|p-q\right\|\right)$) between the pixel locations p and q. $G_{\sigma_{r}}$ is the weight assigned to the depth from point q relative to point p, with $G_{\sigma_{r}}\left(\left|I_{q}\right|\right)$ inversely proportional to the distance value. Similarly, the dense range map can be obtained. In these generated maps, the 2D image intensities are not arbitrary values but rather indicate normalized X, Y, and Z coordinates of 3D space, respectively.

**Figure 3:**
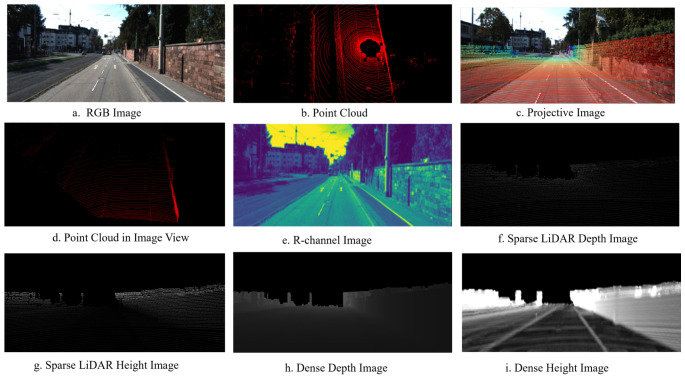
Explanation of pseudo-LiDAR-image generation, where the following are illustrated as follows: (**a**). RGB image; (**b**). raw point cloud; (**c**). projective image; (**d**). point cloud in image FOV; (**e**). R-channel image; (**f**). corresponding sparse LiDAR depth image; (**g**). corresponding sparse LiDAR height image; (**h**). corresponding dense LiDAR depth image; (**i**). corresponding dense LiDAR height image.

Figure 3 presents a comprehensive set of pseudo-LiDAR-Image data representations, Figure 3a represents the cropped RGB image, the LiDAR captures 3D data in Figure 3b presents spatial information of the surrounding environment, reflecting the geometry and structure of objects. Figure 3c is projective points; warm-colored points indicate shorter distances, while cold-colored points correspond to longer distances. Figure 3d is 3D data onto the RGB image’s coordinate system. Figure 3e isolates the red channel from the RGB image. Figure 3f is the sparse depth map. Similarly, Figure 3g focuses on the height (z-coordinate) information, reflecting the vertical distribution; the background appears darkest on the height map, while obstacles are brighter. Figure 3h offers continuous depth information across the entire image plane, while Figure 3i emphasizes the dense height distribution of the elements. Collectively, as shown in Figure 3, early fusion and data complementation are applied, enabling the integration of complementary visual and geometric information to significantly enhance perception performance.

### 3.2. High-Resolution Feature Fusion (HR2FNet)

Standard HRNet is originally designed for single-modal RGB images and lacks an explicit cross-stage interaction mechanism for multi-modal features. When directly applied to our 6-channel pseudo-LiDAR-image data, two critical problems emerge: (1) Input Layer Mismatch. Standard HRNet only accepts 3-channel RGB inputs and cannot directly process pseudo-LiDAR-Image data, which leads to feature dimension mismatch and information loss of geometric cues. (2) Cross-stage Feature Gap. The fixed fusion structure of HRNet only performs alignment between resolution branches, which fails to model the explicit cross-modal interaction between RGB features and LiDAR geometric features, leading to underutilization of multi-modal data.

To address these issues, the proposed HR2FNet modified the input layer to support 6-channel pseudo-LiDAR-Image data as input. It consists of four stages, Stages I to III follow the HRNet backbone, maintaining multi-resolution parallel branches with a cross-stage feature fusion module to preserve high-resolution details. After the pooling layer, Stage IV is indicated as a dilated multi-scale feature fusion module that leverages dilated convolutions to enlarge the receptive field and aggregate multi-scale confidence scores for pixel-wise classification.

#### 3.2.1. HR-Net Branch Fusion Module

Followed by the HRNet [42] backbone, the proposed HR-Net branch module features a parallel multi-resolution branch architecture and enforces repeated multi-scale fusions across these branches. It consists of a three-parallel-branches architecture: a high-resolution (HR) feature branch (main), a moderate-resolution (MR) feature branch, and a low-resolution (LR) feature branch. More specifically, three branches are progressively combined to form Stage I–III, which follow a hierarchical backbone structure to extract multi-scale features from pseudo-LiDAR image input.

Stage I extracts edges and spatial size from different domains in the HR branch. Stage II receives Stage I input, feature maps from HR subnetwork, and enriches representations via a cross-stage feature fusion module, fusing shallow and moderately deep features to preserve spatial info. Stage III further takes the feature maps from the above subnetworks as input, and refines features, leveraging the cross-stage feature fusion module to capture multi-level semantics, from fine details to abstract concepts, building a hierarchical feature pyramid. Followed by HRNet, the residual Basic Block and bottleneck are chosen as building blocks. It consists of two 3 × 3 convolutional layers, each followed by a batch normalization layer and an activation function.

#### 3.2.2. Cross-Stage Feature Fusion Module

**Figure 4:**
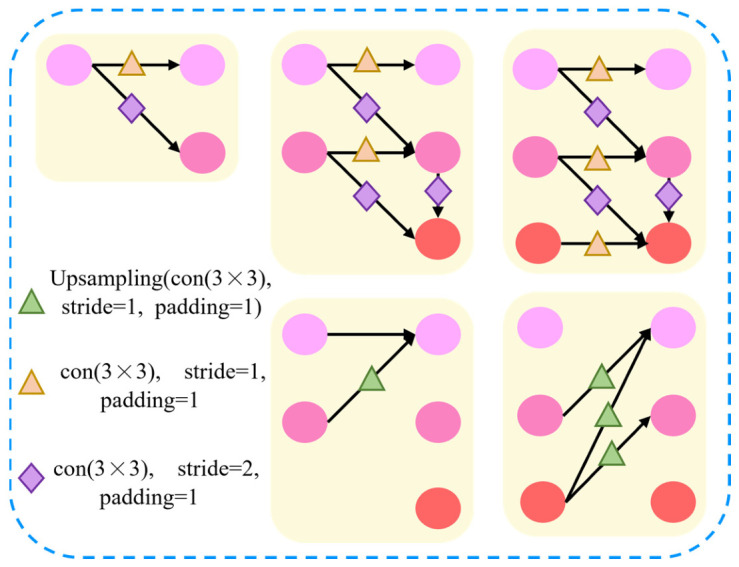
Schematic of cross-stage feature fusion module, corresponding to the yellow part of Figure 2. The circles denote the feature maps in each branch, where light pink, medium pink, and red circles correspond to feature maps of high, medium, and low spatial resolutions, respectively.

To address the cross-stage feature gap between high- and low-resolution branches, the proposed cross-stage feature fusion module is designed to simplify the up/downsampling process and realize efficient feature transfer across different scale branches (schematic shown in Figure 4). The module includes two core steps: (1) downsampling and transferring high-resolution feature maps to low-resolution branches; (2) upsampling and transferring low-resolution feature maps to high-resolution branches. The feature fusion is realized by element-wise addition via a 3×3 convolutional layer, which effectively enhances the information interaction between different branches.

Specifically, feature maps of the same resolution are directly copied. For fusion from high-resolution to low-resolution branches, one or more stride convolutions (stride = 2) are first applied to downsample high-resolution features to corresponding low resolution (a progressive process), and then all feature maps are concatenated along the channel dimension.

Similarly, for fusion from low-resolution to high-resolution branches, bilinear upsampling is performed to resize the low-resolution maps to match the spatial size of the high-resolution features, and a 1×1 convolutional layer is used to unify the number of channels of the feature maps. The final feature fusion is implemented via channel concatenation. The cross-stage feature fusion module aggregates multi-scale feature maps from all branches and transfers cross-scale features back to the high-resolution main branch, which enables the parallel branches to capture rich contextual information and effectively addresses the feature gap between different resolutions.

#### 3.2.3. Dilated Feature Collection Module

It is pertinent to highlight that the stage is designed to aggregate multi-scale confidence scores for precise pixel-wise road/non-road classification, and three parallel convolutional pathways are deployed. In each pathway, only kernel sizes of one 1×1 and two 3×3 convolutions with various dilation rates (R=1,3,5) are applied to generate a two-channel feature map (road/non-road) across multiple scales. Subsequently, an upsampling layer is employed to upscale two-channel feature maps to match spatial resolution. “conv” indicates a convolutional layer; the lightweight decoder is composed of Basic Block and upsampling. At the end of each pathway, three confidence maps {$C_{h}^{0},C_{h}^{1}$} are obtained, where h=1,2,3 is index of pathway. The final confidence map is defined as the average of three confidence maps.

(6) $$C^{h}=\frac{1}{3}\sum_{h=1}^{3}C_{h}^{k},\;k=0,1$$

The dilated feature collection module outputs two confidence maps {$C^{0},C^{1}$}, where $C^{0}$ indicates non-road, and $C^{1}$ for the road region. By leveraging dilated convolutions to enlarge receptive fields of intermediate feature maps, it enables each position to capture all the pixels, facilitating the network to incorporate broader contextual cues that are essential for distinguishing between drivable and non-drivable regions, thereby enhancing the model’s ability to collect global spatial information. Details of network architecture can be found in reference [43,44].

## 4. Experiment

### 4.1. Dataset and Metrics

The proposed LC-HR2FNet is evaluated on the **KITTI Road benchmark** [41], a widely recognized dataset for road segmentation in autonomous driving, developed in collaboration with the Honda Research Institute Europe GmbH (Offenbach am Main, Germany). The benchmark includes four distinct urban road scenarios: Urban Unmarked Road (UU), Urban Marked Road (UM), Urban Multiple Marked Lanes (UMM), and a combined Urban Road scenario (URBAN). The dataset provides synchronized sensor data, including RGB images (default resolution set to 1242×375), Velodyne 64E LiDAR point clouds, sensor calibration parameters, and pixel-level ground-truth labels for road segmentation.

For road segmentation tasks, the KITTI Road dataset is partitioned into a training set (289 samples with ground truth) and a test set (290 samples without ground truth). In this experiment, we only retain the “road” label and reclassify all the other classes as “non-road” to focus on the core road segmentation task. The test results are converted to bird’s-eye view (BEV) images with a resolution of 800 × 400 and submitted to the official KITTI evaluation server for quantitative evaluation. All the visualization and quantitative results are obtained from the KITTI benchmark.

The maximum F-measure (*MaxF*) at the pixel level in BEV space is adopted as the primary evaluation metric for road segmentation, which is a standard metric for the KITTI Road benchmark. In addition, several auxiliary metrics are used to evaluate the performance of the model: Precision (*PRE*), Recall (*REC*), Average Precision (*AP*), False Positive Rate (*FPR*), and False Negative Rate (*FNR*). The definitions of the metrics are as follows:

(7) $$Precision=\frac{TP}{TP+FP}\;\;Recall=\frac{TP}{TP+FN}$$

(8) $$AP=\frac{TP+TN}{TP+FP+TN+FN}$$

(9) $$\;\;FPR=\frac{FP}{FP+TN},\;FNR=\frac{FN}{TP+FN}$$

(10) $$\mathrm{MaxF}=max(\frac{2\times Precision\times Recall}{Precision+Recall})=\frac{2TP}{2TP+FP+FN}$$

where *TP* (True Positive) is the number of road pixels correctly classified, *TN* (True Negative) is the number of non-road pixels correctly classified, *FP* (False Positive) is the number of non-road pixels misclassified as road, and *FN* (False Negative) is the number of road pixels misclassified as non-road. Precision reflects the accuracy of road pixel prediction, while Recall reflects the completeness of road region detection. The *MaxF* metric comprehensively considers both Precision and Recall and is thus the most representative metric for evaluating road segmentation performance [33].

### 4.2. Implementation Details

This model is trained and evaluated on the official KITTI-Road dataset. The training set is further split into a training split (253 samples) and a validation split (36 samples) for model tuning and ablation studies. To focus on the relevant road area and reduce computational cost, the original RGB images 1392×512 are preprocessed with a resolution of 1242×375, and are used for model training and testing.

The proposed approach is implemented based on PyTorch 1.6 and trained on an Ubuntu 18.04 system equipped with an NVIDIA GeForce RTX 3090 GPU and 128 GB of memory.

**Parameter setting basis:** For the baseline model, all key parameters are determined based on network structure characteristics, task demand, and hardware constraints: (1) Dilation rates—For the dilated feature collection module, the rates are set to (1, 3, 5) to capture multi-scale receptive fields. The combination balances fine-grained road boundary details and global road context and avoids the grid artifacts caused by large continuous dilated rates. (2) Training hyperparameters—Learning rate of 0.001, weight decay of 0.001, batch size of 4 are configured to adapt to a 6-channel multimodal feature distribution. These parameter settings ensure balanced training stability, suppress overfitting, and balance gradient optimization quality and GPU memory limitations.

**Parameter sensitivity Analysis:** Single-variable ablation experiments on the KITTI validation set are conducted. The results verify that our parameter settings achieve optimal performance and stability. Improper parameter configurations lead to insufficient context extraction, feature artifacts, or unstable training. These validations fully prove the rationality and robustness of our hyperparameter design, improving the reproducibility.

### 4.3. Experimental Results

#### 4.3.1. Ablation Study on Input Modalities and Network Modules

To verify the performance contribution of different input modalities and network modules, two sets of ablation experiments are conducted on the KITTI validation set: (1) comparison of segmentation performance with different input modalities (RGB-only, LiDAR-only and pseudo-LiDAR-Image) and (2) comparison of performance with different combinations of network modules (HRNet-Branch fusion, Cross-stage feature fusion, dilated feature collection).

**Ablation on Input Modalities:** Table 1 presents the segmentation performance of the model with three different input modalities on the KITTI validation set. Notably, when employing Pseudo LiDAR-Image data, the network input channels are configured to 6 (3 RGB channels + 3 LiDAR channels), while RGB-only and LiDAR-only use three-channel inputs.

As can be seen in Table 1, the results show that the fusion of LiDAR and camera data into the pseudo-LiDAR-Image representation yields significant performance improvements in all metrics (MaxF, AP, PRE, and REC), with an improvement of 1–3% over single-modality inputs. The model with pseudo-LiDAR-Image input achieves a *MaxF* score of 96.76%, which is 1.45% higher than the RGB-only baseline and 1.18% higher than the LiDAR-only baseline. This demonstrates that the proposed early-level fusion strategy effectively integrates the complementary visual and geometric information of LiDAR and camera, and the pseudo-LiDAR-Image representation provides a more comprehensive feature set for road segmentation.

**Figure 5:**
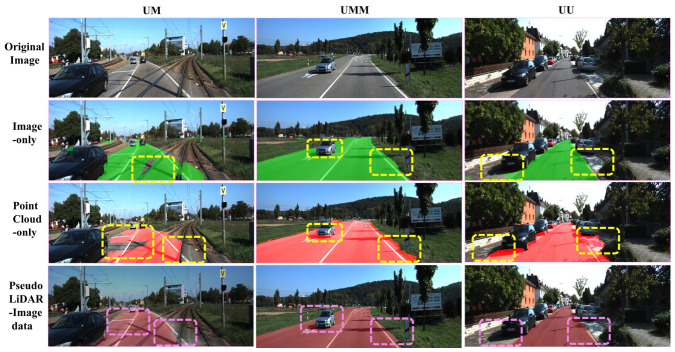
Visualization results for model prediction by different inputs. From left to right in the first row, there are UM, UMM, and UU scenarios. The colored dashed boxes are used to highlight typical regions for visual comparison.

Figure 5 shows the visualization results of the model with different input modalities on the KITTI validation set. It can be seen that the RGB-only model suffers from severe prediction hollowing in road regions affected by uneven texture and heavy shadows (second row in Figure 5). The LiDAR-only model is less sensitive to illumination variations and achieves better performance, but it still has misclassification in vehicle boundary and roadside regions due to the sparsity of point clouds. In contrast, the model with pseudo-LiDAR-Image input largely corrects the misclassified regions in single-modality predictions and accurately segments the road boundary even in complex scenarios. This compellingly demonstrates that multi-modal fusion enhances road segmentation performance by effectively leveraging complementary cues from both LiDAR and camera data.

**Ablation on network modules**: Table 2 presents the segmentation performance of the model with different combinations of network modules, where the best performance is marked in bold.

The results show that each module contributes to the performance improvement of the model: (1) the basic HRNet-Branch fusion module achieves a MaxF of 95.08%; (2) adding the Cross-stage feature fusion module increases the MaxF by 0.83% to 95.91%, which validates the effectiveness of cross-scale feature interaction; (3) adding the dilated feature collection module to the basic HRNet-Branch fusion module increases the MaxF by 1.27% to 96.35%, which demonstrates the advantage of multi-scale confidence aggregation; (4) the model with all three modules achieves the optimal MaxF of 96.76%, which is the best performance in all combinations.

This confirms that the proposed network modules are complementary and their combination can significantly improve the road segmentation performance of the model.

**Figure 6:**
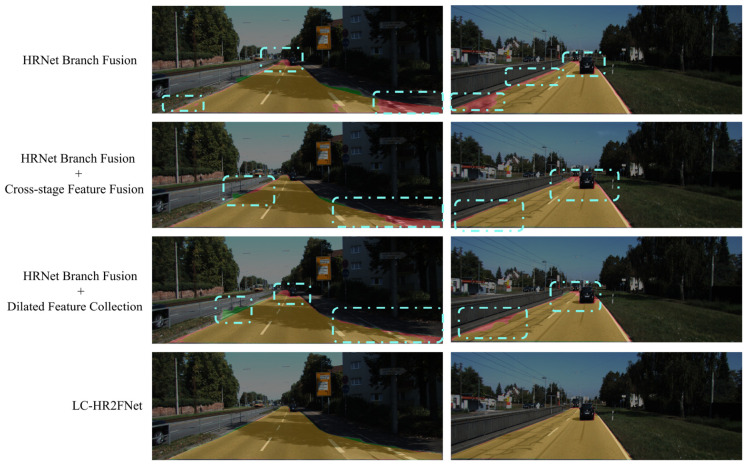
Visualization results for model prediction with various settings. From top to bottom are HRNet branch-Fusion based, HRNet branch with Cross-stage module-based, HRNet branch with dilated feature collection-based and the proposed HR2FNet in UM, scenarios. The colored dashed boxes are used to highlight typical regions for visual comparison.

Figure 6 shows the visualization results of the model with different module combinations on the UM scenario, where yellowish-brown denotes road regions, red denotes missing road pixels (*FN*), and green denotes misclassified non-road pixels (*FP*).

The results show that the baseline HRNet-Branch fusion model produces noticeable false positive predictions in complex scenes with occlusions and shadows, and has line boundary errors at the roadside (due to the similar texture and structure of the roadside and pavement). The integration of the cross-stage feature fusion module refines the feature representation and leads to more coherent road boundaries, reducing the number of boundary errors. The dilated feature collection module enhances the model’s ability to capture multi-scale contextual information, significantly reducing the number of spurious detections. In contrast, the proposed LC-HR2FNet delivers the most accurate segmentation results, effectively delineating driven road regions even in the presence of vehicles and roadside obstacles.

#### 4.3.2. Evaluation on KITTI Road Benchmark Test Dataset

For a comprehensive evaluation of the proposed method, the LC-HR2FNet is tested on the unlabeled KITTI Road test set, and prediction results are submitted to the official evaluation server for quantitative analysis. The BEV region evaluated in the experiment corresponds to an area approximately 10 m ahead (6–46 m) and 10 m to each side of the vehicle, which is the key perceptual region for autonomous driving. Table 3 presents the quantitative performance of the proposed model on the four KITTI Road scenarios (UM_ROAD, UMM_ROAD, UU_ROAD, URBAN_ROAD), obtained directly from the official evaluation server.

The proposed method achieves a Max F score of 97.39% on the UMM_ROAD dataset and an average MaxF of 93.70% across all urban scenarios. Among the tested scenarios, the UU_ROAD scenario exhibits the lowest performance, which is attributed to its complex and varied environmental conditions, as well as more irregular road characteristics compared to the other scenarios in the dataset.

**Figure 7:**
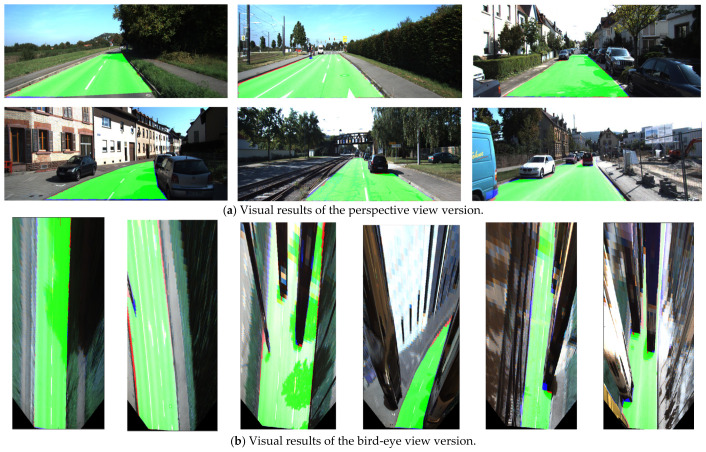
Perspective and bird-eye view visualization results. (**a**) is perspective view converted into BEV, while the green-colored area in (**b**) represents road area detected by using proposed method.

Figure 7 presents the visualization results on the test dataset, including perspective view segmentation results (Figure 7a) and BEV segmentation results (Figure 7b). Each row of perspective view corresponds to the same scene in the BEV. Perspective view results should be transformed to BEV view, which facilitates the observation of road boundary segmentation effects (distant pixels are more important in the BEV view due to the decreasing resolution of the perspective view with distance). In visualization results, green pixels denote correctly classified road regions (*TP*), red pixels denote incorrect road regions (*FP*), and blue pixels denote missing road regions (*FN*). The results show that the proposed LC-HR2FNet accurately segments the road regions even in complex scenarios with vehicles, shadows, and roadside obstacles, and the road boundaries are clearly delineated in both perspective and BEV. This demonstrates the robust performance of the model for road segmentation in real autonomous driving environments.

#### 4.3.3. Experiments with Recent Methods

To further validate the effectiveness of proposed LC-HR2FNet, we compare it with previous methods on KITTI Road benchmark. First, comparison results on UM_ROAD scenario are presented in Table 4. Next, the performance on UMM_ROAD scenario is reported in Table 5. The results for UU_ROAD scenario are shown in Table 6, and the overall performance on the URBAN_ROAD scenario is summarized in Table 7.

The methods compared include RESD-Velo, PLARD, LidCamNet, FusedCRF, HybridCRF, MixedCRF, Lightfusion, and Epurate-Net. These methods cover different fusion strategies (early-level, middle-level, late-level) and network architecture, and are the most representative methods for LiDAR-camera fusion-based road segmentation.

As illustrated in Table 4, Table 5, Table 6 and Table 7, the proposed LC-HR2FNet achieves competitive performance across all four scenarios; it ranks third in Urban_Road with *MaxF* (96.28%), *PRE* (96.76%), and *REC* (96.58%). It outperforms traditional CRF-based methods (FusedCRF, HybridCRF, MixedCRF) and the early LiDAR-based method (RES3D-Velo) by a large margin (*MaxF* improvement of 5–15%), which validates the advantage of the deep learning-based high-resolution fusion framework over traditional methods.

Compared with the lightweight fusion method (LightFusion), LC-HR2FNet achieves a *MaxF* improvement of 1.02% on the Urban scenario and a PRE improvement of 0.64%, which confirms the robustness and accuracy of the proposed method in diverse urban road environments.

Note that the PLARD that has a *MaxF* of 97.03% is trained on multiple datasets, while this method is only trained on the KITTI dataset. Epurate-Net constructs cross-layer attention to aggregate global–local contextual information with several rounds. Moreover, PLARD, LidCamNet, and Epurate-Net adopt middle-level fusion, which involves multiple rounds of feature refinement within deep network layers. In contrast, our method focuses on an early-level fusion strategy and interaction between multi-stage multi-scale features. This design choice simplifies computational complexity while still ensuring effective cross-modal information integration, offering a favorable trade-off between performance and efficiency.

The proposed method achieves slightly lower *MaxF* scores than PLARD and EpurateNet on some scenarios, but it is important to note that: (1) PLARD is trained on multiple datasets, while our method is only trained on the KITTI dataset; (2) EpurateNet constructs cross-layer attention to aggregate global–local contextual information with multiple rounds of refinement, which significantly increases the computational complexity; (3) PLARD, LidCamNet, and EpurateNet all adopt middle-level fusion strategies with multiple rounds of feature refinement in deep network layers, while our method focuses on an early-level fusion strategy with cross-stage multi-scale feature interaction, which simplifies the computational complexity while ensuring effective cross-modal information integration.

## 5. Conclusions

This paper proposes LC-HR2FNet, a LiDAR-camera high-resolution feature fusion network to address the problems of insufficient contextual information and inadequate multimodal fusion due to modality gaps. A multi-cross-stage fusion framework is designed. An early fusion strategy is adopted to generate dense pseudo-LiDAR-image data, which complements sparse point clouds with RGB images to bridge the modality gap. A modified HRNet with cross-stage feature fusion is constructed to maintain high-resolution details and enhance inter-branch information interaction. Meanwhile, a dilated feature collection module is introduced to capture multi-scale contextual information for reliable pixel-wise classification.

**Despite the promising performance, our work still has some limitations:**

1. This method relies on high-precision LiDAR-Camera calibration. Severe misalignment (more than 3 px/3°) will cause significant performance degradation, which requires regular recalibration in real applications.
2. The dense data complementation step slightly increases the preprocessing time, which brings a small overhead to the system latency.
3. We have only tested this method on the KITTI dataset; the generalization ability to extreme weather conditions (rain, fog, snow) still needs further verification.

Based on the above limitations, we will further explore the following directions in **future work:**

1. Develop lightweight fusion modules to reduce the preprocessing latency to meet the real-time requirements of autonomous driving systems.
2. Introduce anti-misalignment calibration and robust feature extraction modules to improve the robustness of our method against sensor misalignment and reduce the dependence on high-precision calibration.
3. Extend our method to adverse weather datasets and real-vehicle platforms to improve the generalization ability of our model in a real-world environment.

## Figures and Tables

**Table 1:** Comparison results under different modalities (/%).

Modality Input Data	*MaxF*	*AP*	*PRE*	*REC*
RGB-only	95.31	92.36	95.24	96.03
3D LiDAR data-only	95.58	93.01	95.83	96.43
Pseudo LiDAR-Image data	96.76	95.13	96.21	97.21

**Table 2:** Comparisons of settings with different modules. The best is bold.

HRNet-Branch Fusion	Cross-Stage Feature Fusion	Dilated Feature Collection	*MaxF* (%)	*AP* (%)	*PRE* (%)	*REC* (%)
√			95.08	92.36	95.24	96.03
√	√		95.91	94.57	95.33	96.64
√		√	96.35	94.55	95.49	96.71
**√**	**√**	**√**	**96.76**	**95.13**	**96.21**	**97.21**

**Table 3:** Performance of the proposed model on KITTI (BEV).

Dataset	*MaxF*	*AP*	*PRE*	*REC*	*FPR*	*FNR*
UM_ROAD	96.01	93.15	95.74	96.05	3.26	4.37
UMM_ROAD	97.39	93.45	97.11	97.39	5.53	4.59
UU_ROAD	95.44	94.52	96.14	96.31	2.58	8.23
URBAN_ROAD	96.28	93.70	96.33	96.58	3.79	5.73

**Table 4:** Comparison with several popular fusion-based methods on UM_ROAD (BEV) (/%).

Method	*MaxF*	*AP*	*PRE*	*REC*	*FPR*	*FNR*
PLARD	97.05	93.53	97.18	96.92	1.28	3.08
LidCamNet	95.62	93.54	95.77	95.48	1.92	4.52
RES3D-Velo	83.81	73.95	78.56	89.80	11.16	10.20
FusedCRF	89.55	80.00	84.87	94.71	7.70	5.22
HybridCRF	90.99	85.26	90.65	91.33	4.29	8.67
MixedCRF	91.57	84.68	90.02	93.19	4.71	6.81
LightFusion	96.03	93.35	95.45	96.63	-	-
Epurate-Net	96.39	-	95.75	97.05	-	-
Ours	96.61	93.15	95.74	96.05	3.26	4.37

**Table 5:** Comparison with several popular fusion-based methods on UMM_ROAD (BEV) (/%).

Method	*MaxF*	*AP*	*PRE*	*REC*	*FPR*	*FNR*
PLARD	97.77	95.64	97.75	97.79	2.48	2.21
LidCamNet	97.08	95.51	97.28	96.88	2.98	3.12
RES3D-Velo	90.60	85.38	85.96	95.78	17.20	4.22
FusedCRF	89.51	83.53	86.64	92.58	15.69	7.42
HybridCRF	91.95	86.44	94.01	89.98	6.30	10.02
MixedCRF	92.75	90.24	94.03	91.50	6.39	8.50
LightFusion	97.69	95.57	97.42	97.96	-	-
Epurate-Net	97.91	-	97.75	98.08	-	-
Ours	97.39	93.45	97.11	97.39	5.53	4.59

**Table 6:** Comparison with several popular fusion-based methods on UU_ROAD (BEV) (/%).

Method	*MaxF*	*AP*	*PRE*	*REC*	*FPR*	*FNR*
PLARD	95.95	95.25	96.25	95.65	1.21	4.35
LidCamNet	94.54	92.74	94.64	94.45	1.74	5.55
RES3D-Velo	83.63	72.58	77.38	90.97	8.67	9.03
FusedCRF	84.49	72.35	77.13	93.40	9.02	6.60
HybridCRF	88.53	80.79	86.41	90.76	4.65	9.24
MixedCRF	85.69	75.12	80.17	92.02	7.42	7.98
LightFusion	95.04	92.61	94.22	95.88	-	-
Epurate-Net	96.44	-	95.89	97.00	-	-
Ours	95.44	94.52	96.14	96.31	2.58	8.23

**Table 7:** Comparison with several popular fusion-based methods on URBAN_ROAD (BEV) (/%).

Method	*MaxF*	*AP*	*PRE*	*REC*	*FPR*	*FNR*
PLARD	97.03	94.03	97.19	96.88	1.54	3.12
LidCamNet	96.03	93.93	96.23	95.83	2.07	4.17
RES3D-Velo	86.58	78.34	82.63	90.92	10.53	9.08
FusedCRF	88.25	79.24	83.62	93.44	10.08	6.56
HybridCRF	90.81	86.01	91.05	90.57	4.90	9.43
MixedCRF	90.59	84.24	89.11	92.13	6.20	7.87
LightFusion	95.26	93.84	95.69	96.82	-	-
Epurate-Net	97.09	93.08	96.76	97.43	1.89	2.76
Ours	96.28	93.70	96.33	96.58	3.79	5.73

## Data Availability

The datasets generated during the current study are available from the corresponding author upon reasonable request.
